# S3‐Leitlinie zur Therapie der Psoriasis vulgaris, adaptiert von EuroGuiDerm – Teil 2: Hilfestellungen für besondere klinische Situationen und bei Vorliegen von Komorbiditäten

**DOI:** 10.1111/ddg.16001_g

**Published:** 2026-02-05

**Authors:** Alexander Nast, Andreas Altenburg, Matthias Augustin, Frank Bachmann, Wolf‐Henning Boehncke, Markus Cornberg, Hilte Geerdes‐Fenge, Brit Häcker, Peter Härle, Joachim Klaus, Michaela Köhm, Arno Köllner, Ulrich Mrowietz, Hans‐Michael Ockenfels, Antonia Pennitz, Sandra Philipp, Thomas Richter, Thomas Rosenbach, Tom Schaberg, Martin Schlaeger, Gerhard Schmid‐Ott, Michael Sebastian, Karisa Thölken, Ralph von Kiedrowski, Uwe Willuhn, Christoph Zeyen

**Affiliations:** ^1^ Division of Evidence‐Based Medicine (dEBM) Klinik für Dermatologie Venerologie und Allergologie Charité ‐ Universitätsmedizin Berlin corporate member of Freie Universität Berlin and Humboldt‐Universität zu Berlin Berlin Deutschland; ^2^ Hochschulklinik für Dermatologie Venerologie und Allergologie Städtisches Klinikum Dessau Dessau Deutschland; ^3^ Institut für Versorgungsforschung in der Dermatologie und bei Pflegeberufen (IVDP) Universitätsklinikum Hamburg‐Eppendorf (UKE) Hamburg Deutschland; ^4^ Hautzentrum Berlin Siddi & Bachmann Berlin Deutschland; ^5^ Service de Dermatologie et Vénéréologie Hôpitaux Universitaires de Genève Genf Schweiz; ^6^ Klinik für Gastroenterologie Hepatologie und Endokrinologie an der Medizinischen Hochschule Hannover Hannover Deutschland; ^7^ Klinik für Infektiologie und Tropenmedizin Department für Innere Medizin Universitätsmedizin Rostock Rostock Deutschland; ^8^ Deutsches Zentralkomitee zur Bekämpfung der Tuberkulose (DZK) Berlin Deutschland; ^9^ Rheumatologisches Zentrum nach G‐BA Klinik für Rheumatologie Klinische Immunologie und Physikalische Therapie Marienhaus Klinikum Mainz Mainz Deutschland; ^10^ Deutscher Psoriasis Bund Hamburg Deutschland; ^11^ Abteilung Rheumatologie Immunologie ‐ Entzündungsmedizin Universitätsklinikum Frankfurt Goethe‐Universität Frankfurt am Main Deutschland; ^12^ Fraunhofer Institut für Translationale Medizin und Pharmakologie ITMP Frankfurt Frankfurt am Main Deutschland; ^13^ Hautarztpraxis Duisburg Deutschland; ^14^ Psoriasis‐Zentrum Klinik für Dermatologie Venerologie Allergologie Universitätsklinikum Schleswig‐Holstein Campus Kiel Kiel Deutschland; ^15^ Haut‐ und Allergieklinik Klinikum Hanau Hanau Deutschland; ^16^ Hautarztpraxis Oranienburg Deutschland; ^17^ Helios Versorgungszentren GmbH MVZ Gotha Gotha Deutschland; ^18^ Hautarztpraxis Osnabrück Deutschland; ^19^ Hautarztpraxis Oldenburg Deutschland; ^20^ Berolina Klinik Löhne Deutschland; ^21^ Hautarztpraxis Mahlow Deutschland; ^22^ Universitätsklinik für Dermatologie Universitätsklinikum Augsburg Augsburg Deutschland; ^23^ Hautarztpraxis Selters Deutschland

**Keywords:** Empfehlungen, Hepatitis, Komorbidität, Psoriasis, Tuberkulose, Comorbidity, hepatitis, psoriasis, recommendations, tuberculosis

## Abstract

Der vorliegende Teil 2 der aktualisierten S3‐Leitlinie zur Therapie der Psoriasis vulgaris bietet Empfehlungen zur Therapieauswahl in besonderen klinischen Situationen sowie bei Vorliegen von Komorbidität. Ein Schwerpunkt des Updates liegt im Kapitel Screening auf Tuberkulose sowie in der Therapieauswahl und im Management bei latenter Tuberkulose. Die Empfehlungen zum Einsatz des Interferon‐Gamma‐Release‐Assays und zur Indikation einer Thoraxröntgenaufnahme wurden umfassend überarbeitet. Ebenso wurden die Aussagen zur Eignung systemischer Psoriasistherapien bei latenter Tuberkulose sowie zur Notwendigkeit einer präventiven antituberkulösen Therapie grundlegend aktualisiert. Im Kapitel entzündliche Darmerkrankungen wurden Risankizumab und Guselkumab als empfohlene Therapieoptionen ergänzt, da beide Substanzen mittlerweile auch für die Indikationen Morbus Crohn und Colitis ulcerosa zugelassen sind. Weitere wesentliche Anpassungen betreffen die Kapitel Patienten mit Malignomen in der Vorgeschichte sowie Virushepatitis.

## HINWEISE ZUR ANWENDUNG DER LEITLINIE

Diese Publikation beinhaltet ausgewählte Kapitel und Textpassagen, in denen besonders relevante Änderungen vorgenommen wurden. Neben diesen hier in Teil 2 dargestellten Abschnitten, enthält Teil 1 unter anderem die Kapitel „Schweregrad und Therapieziele“, „Einleitung und Auswahl einer systemischen Therapie“, „Übersicht der Therapieoptionen“, „Ergebnisse der Netzwerk‐Metaanalyse“ sowie die „Anwendungshinweise zu den einzelnen Medikamenten“

Die Langfassung der Leitlinie befindet sich auf den Seiten der AWMF (https://register.awmf.org/de/leitlinien/detail/013‐001). Insbesondere die Informationen des Kapitels „Hinweise zur Anwendung der Leitlinie/Haftungsausschluss“ der Langfassung sind für die Anwendung der in dieser Kurzfassung dargestellten Leitlinienempfehlungen zu beachten. Auf den Seiten der AWMF befinden sich auch folgende Begleitdokumente zur Version 8 der Leitlinie: Appendix A („Empfehlungen zu topischer Therapie“, „Phototherapie“, „sonstige Therapien“, „Schnittstellendefinition“), Evidenzbericht, Leitlinienreport mit Angaben zu Interessenkonflikten, PowerPoint Foliensatz zur Leitlinienimplementierung.

*Für den Abschnitt „Hinweise zur Anwendung der Leitlinie/Haftungsausschluss“ (diese gelten in gleichem Maße für die vorliegenden Kurzfassungen) siehe Langfassung*.

## HILFESTELLUNGEN FÜR BESONDERE KLINISCHE SITUATIONEN UND BEIM VORLIEGEN VON KOMORBIDITÄTEN

Tabelle 1 und 2 geben einen Überblick über die ausgewählten besonderen klinischen Entscheidungssituationen und die Einschätzung der Leitliniengruppe zu diesen Medikamenten. Tabelle 3 erläutert die Farben und Symbole der Tabellen 1 und 2.

**TABELLE 1:** Entscheidungsmatrix (I) zu den konventionellen Therapien mit Expertenkonsens zu deren Eignung in besonderen klinischen Situationen.

	**Konventionelle Systemtherapeutika**			
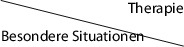	**Acitretin**	**Ciclosporin**	**Fumarate**	**Methotrexat**
**Chronisch entzündliche Darmerkrankung: Morbus Crohn**	**↑** insbesondere Fälle mit leichter paradoxer Psoriasis			**↑**
**Chronisch entzündliche Darmerkrankung: Colitis ulcerosa**	**↑** insbesondere Fälle mit leichter paradoxer Psoriasis	**↑**		
**Diabetes mellitus / metabolisches Syndrom**		Alternativen in Betracht ziehen		Alternativen in Betracht ziehen
**Dyslipidämie**	**↓**	**↓**		
**Fortgeschrittene Herzinsuffizienz**	**↑**	**↓**		**↑**
**Herzkrankheit: Ischämische Herzerkrankung (KHK)**	**↓**	**↓**		**↑**
**latente TB‐Infektion / behandelte Tuberkulose**	**↑↑**	**↑↑**	**↑↑**	**↑↑**
**Schwangerschaft**	**↓↓**		**↓**	**↓↓**

**TABELLE 2:** Entscheidungsmatrix (II) zu den Biologika und neuen zielgerichteten kleinen Molekülen mit Expertenkonsens zu deren Eignung in besonderen klinischen Situationen.

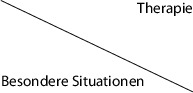	PDE‐4‐Inhibitor	TYK‐2 Inhibitor	TNF‐Inhibitoren				IL12/23p40‐Inhibitoren	IL17‐Inhibitoren				IL23‐Inhibitoren		
	Apremilast	Deucravacitinib	Etanercept	Infliximab	Adalimumab	Certolizumab	Ustekinumab	Secukinumab	Ixekizumab	Brodalumab	Bimekizumab	Guselkumab	Tildrakizumab	Risankizumab
**Chronisch entzündliche Darmerkrankung: Morbus Crohn**				**↑↑**				**↓**				**↑↑**	**↑**	**↑↑**
**Chronisch entzündliche Darmerkrankung: Colitis ulcerosa**	**↑**			**↑↑**			**↑↑**	**↓**				**↑↑**	**↑**	**↑↑**
**Diabetes mellitus/metabolisches Syndrom**														
**Dyslipidämie**														
**Fortgeschrittene Herzinsuffizienz**	**↑**		**↓**				**↑**							
**Herzkrankheit: KHK**			**↑**											
**latente TB‐Infektion/behandelte Tuberkulose**	**↑↑**	**↓**	**↓↓**				**↑**							
**Schwangerschaft**	**↓**	**↓**				**↑**								

**TABELLE 3:** Legende für Tabelle 1 und Tabelle 2.

Symbole	Bedeutung
**↑↑**	Wir sind der Auffassung, dass alle oder fast alle informierten Menschen diese Entscheidung treffen würden. Klinikeren müssen sich weniger Zeit für den Prozess der Entscheidungsfindung mit den Patienten nehmen. In den meisten klinischen Situationen kann die Empfehlung als allgemeine Vorgehensweise übernommen werden.
**↑**	Wir sind der Auffassung, dass die meisten informierten Menschen, ein substanzieller Anteil jedoch nicht, diese Entscheidung treffen würden. Kliniker und andere Anbieter von Gesundheitsleistungen müssen mehr Zeit aufwenden, um sicherzustellen, dass die Wahl des Verfahrens mitsamt der möglicherweise verbundenen Konsequenzen die Werte und Präferenzen der individuellen Patienten widerspiegelt. Entscheidungsprozesse im Gesundheitssystem erfordern eine tiefgehende Diskussion und die Einbeziehung vieler Stakeholder.
	Siehe Hintergrundtext und spezifische Empfehlungen im jeweiligen Kapitel
**↓**	Wir sind der Auffassung, dass die meisten informierten Menschen, ein substanzieller Anteil jedoch nicht, eine Entscheidung gegen diese Intervention treffen würden.
**↓↓**	Wir sind der Auffassung, dass alle oder fast alle informierten Menschen eine Entscheidung gegen diese Intervention treffen würden. In den meisten klinischen Situationen kann die Empfehlung als allgemeine Vorgehensweise übernommen werden.

### Übersicht der Themengebiete und zu Behandelnden Fragen Bei Vorliegen Von Komorbidität und für Besondere Klinische Situationen

**Table:** 

**THEMENGEBIET**	**FRAGEN**
Chronisch entzündliche Darmerkrankungen	Wie sollte eine Psoriasis bei Patienten mit gleichzeitigem Vorliegen einer chronisch entzündlichen Darmerkrankung behandelt werden?
Krebs	Wie sollte eine Psoriasis bei Patienten mit Malignomen in der Vorgeschichte behandelt werden?
Depression	Wie soll eine Psoriasis bei Patienten mit Depressionen und/oder Suizidgedanken behandelt werden?
Diabetes mellitus	Wie soll eine Psoriasis bei Patienten mit Diabetes mellitus behandelt werden?
Herzkrankheit	Wie soll eine Psoriasis bei Patienten mit KHK und/oder Herzinsuffizienz mit reduzierter Ejektionsfraktion (HFrEF) behandelt werden?
Nierenerkrankung	Wie soll eine Psoriasis bei Patienten mit Nierenversagen/Nierenschädigung behandelt werden?
Neurologische Erkrankungen	Welche Behandlungen sind für Patienten mit Psoriasis und neurologischen Erkrankungen geeignet?
Virushepatitis	Wann und wie sollten Patienten mit Psoriasis auf Virushepatitis untersucht werden, und wie sollten Patienten, die positiv getestet werden, behandelt werden?
Tuberkulose‐Screening	Wie soll vor und während einer Systemtherapie auf Tuberkulose gescreent werden?
Tuberkulose und Therapie	Wie soll das Management bei Patienten mit Psoriasis und einem positivem Interferon Gamma Release Assay (IGRA) erfolgen?
Kinderwunsch und Schwangerschaft	Wie sollten Patienten mit Psoriasis und aktuellem Kinderwunsch oder während einer Schwangerschaft behandelt werden?
Impfungen	Wie sollten Impfungen bei Patienten mit Psoriasis unter systemischer Behandlung gehandhabt werden?

## ENTZÜNDLICHE DARMERKRANKUNGEN: WIE SOLLTE EINE PSORIASIS BEI PATIENTEN MIT GLEICHZEITIGEM VORLIEGEN EINER CHRONISCH ENTZÜNDLICHEN DARMERKRANKUNG BEHANDELT WERDEN?

Nach der öffentlichen Konsultation erfolgte für die Wirkstoffe Risankizumab und Guselkumab eine Neubeurteilung basierend auf dem Zulassungsstatus der Psoriasis‐Therapien für Morbus Crohn und Colitis ulcerosa. Für den erläuternden Hintergrundtext zu diesem Kapitel siehe Langfassung.

**Empfehlungen**:

**Table:** 

3.1‐1 | geprüft [2025] Bei einer systemischen Behandlung der Psoriasis bei Patienten mit einer begleitentenden chronisch entzündlichen Darmerkrankung (CED) *wird* eine Zusammenarbeit mit den behandelnden Gastroenterologen *empfohlen*.	**↑↑**	starker konsens Konsensbasiert
3.1‐2 | modifiziert [2025] Es *wird empfohlen*, bei Patienten mit Psoriasis und einer CED vorzugsweise zugelassene zielgerichtete Therapien mit dokumentierter Wirksamkeit bei diesen Erkrankungen einzusetzen: *Morbus Crohn*: TNFi (Infliximab, Adalimumab, Certolizumab*), anti‐IL‐12/23p40 (Ustekinumab) oder anti‐IL‐23p19 (Risankizumab, Guselkumab) *Colitis ulcerosa*: TNFi (Infliximab, Adalimumab), anti‐IL‐12/23p40 (Ustekinumab) oder anti‐IL‐23p19 (Risankizumab, Guselkumab) ^*^Zum Zeitpunkt der Aktualisierung der Leitlinie zur Version 8 ist Certolizumab pegol in Deutschland nicht für die Indikation des Morbus Crohn zugelassen.	**↑↑**	starker konsens ^1^ Konsensbasiert
3.1‐3 | modifiziert [2025] Wenn die Wirkstoffe der Empfehlung 3.1‐2 nicht eingesetzt werden können, *kann es empfohlen werden*, die folgende Behandlungsoption bei Patienten mit Psoriasis und CED in Betracht zu ziehen: *Morbus Crohn*: Tildrakizumab *Colitis ulcerosa*: Tildrakizumab	**↑**	starker konsens ^1^ Konsensbasiert
3.1‐4 | modifiziert [2025] Wenn die Wirkstoffe der Empfehlung 3.1‐2 nicht eingesetzt werden können, *kann es empfohlen werden*, die folgenden oralen Behandlungsoptionen bei Patienten mit Psoriasis und CED in Betracht zu ziehen: *Morbus Crohn*: Methotrexat *Aktive Colitis ulcerosa*: Ciclosporin (bevorzugt), Apremilast (auch möglich)	**↑**	starker konsens ^1^ Konsensbasiert
3.1‐5 | geprüft [2025] Es *kann empfohlen werden*, in Kombination mit anderen Behandlungen, Acitretin als Zusatztherapie für Patienten mit CED und Psoriasis einzusetzen, insbesondere bei Fällen mit leichter paradoxer Psoriasis.	**↑**	starker konsens ^1^ Konsensbasiert
3.1‐6 | geprüft [2025] Der Einsatz von IL‐17‐Inhibitoren bei Patienten mit entzündlichen Darmerkrankungen *kann nicht empfohlen werden*.	**↓**	starker konsens ^1^ Konsensbasiert

^1^ Sechs Enthaltungen aufgrund von Interessenkonflikten.

## KREBS: WIE SOLLTE EINE PSORIASIS BEI PATIENTEN MIT MALIGNOMEN IN DER VORGESCHICHTE BEHANDELT WERDEN?

Für den erläuternden Hintergrundtext siehe Langfassung.

**Table:** 

3.2‐1 | neu [2025] *Krebserkrankung oder Remission < 5 Jahre* Bei Patienten mit Psoriasis und einer aktuellen oder innerhalb der letzten fünf Jahren erfolgten Krebsdiagnose, *wird empfohlen*, die Entscheidung zur Einleitung einer immunsuppressiven/immunmodulierenden Therapien mit einem/einer auf die Krebsart spezialisierten Arzt/Ärztin zu besprechen und mit den Patienten eine gemeinsame, informierte Entscheidung zu treffen, die die Präferenz der Patienten respektiert. (Bei Tumorentitäten mit dem Risiko einer späten Metastasierung und gegebenenfalls noch fortbestehender Therapie (zum Beispiel Prostata) gilt dies auch über 5 Jahre hinaus.)	**↑↑**	starker konsens Konsensbasiert
3.2‐2 | Neu [2025] *Remission > 5 Jahre* Für Patienten, die sich länger als 5 Jahre in Remission einer Krebserkrankung befinden, *wird empfohlen*, die für die Therapie der Psoriasis am besten geeignete Option unabhängig von der Krebserkrankung auszuwählen. (Für Ausnahmen siehe Empfehlung zur ‚Krebserkrankung oder Remission < 5 Jahre‘)	**↑↑**	starker konsens Konsensbasiert
3.2‐3 | neu [2025] *Palliative Situation* Für Patienten in einer Palliativsituation *wird* eine maximal lebensqualitätserhaltene Therapie in Abstimmung mit den behandelnden Onkologen/Palliativmedizinern *empfohlen*.	**↑↑**	starker konsens Konsensbasiert

*Für die Kapitel „Depression“, „Nierenerkrankung“, „Neurologische Erkrankungen“ siehe Langfassung*.

## DIABETES: WIE SOLL EINE PSORIASIS BEI PATIENTEN MIT DIABETES MELLITUS BEHANDELT WERDEN?

Für den erläuternden Hintergrundtext siehe Langfassung.

**Table:** 

3.4‐1 | Neu [2025] Wenn alternative Behandlungen möglich sind, *kann* bei Patienten mit Diabetes mellitus Typ 2 (bei denen zusätzlich ein metabolisches Syndrom und/oder Zeichen für eine Leberschädigung vorliegen) *empfohlen werden*, Alternativen zu MTX zu verschreiben.	**↑**	starker konsens ^1^ Konsensbasiert
3.4‐2 | Neu [2025] Wenn alternative Behandlungen möglich sind, *kann* bei Patienten mit Diabetes mellitus Typ 2 (bei denen zusätzlich ein metabolisches Syndrom und/oder Zeichen für eine Leberschädigung vorliegen) *empfohlen werden*, Alternativen zu Ciclosporin zu verschreiben.	**↑**	starker konsens ^1^ Konsensbasiert
3.4‐3 | Geprüft [2025] Eine Therapie mit Acitretin oder Ciclosporin als Erstlinienbehandlung bei Patienten mit Dyslipidämie *kann nicht empfohlen werden*.	**↓**	starker konsens ^1^ Konsensbasiert

^1^ Drei Enthaltungen aufgrund von Interessenkonflikten.

## VIRUSHEPATITIS: WANN UND WIE SOLLTEN PATIENTEN MIT PSORIASIS AUF VIRUSHEPATITIS UNTERSUCHT WERDEN, UND WIE SOLLTEN PATIENTEN, DIE POSITIV GETESTET WERDEN, BEHANDELT WERDEN?

Es wurde ein systematischer Review zur Behandlung von Patienten mit Psoriasis und viraler Hepatitis durchgeführt. Einzelheiten hierzu sowie eine narrative Synthese der identifizierten Evidenz entnehmen Sie bitte dem Kapitel 3 des Evidenzberichts.

Zudem erfolgte ein Abgleich mit und eine teilweise Adaptierung der S3‐Leitlinie der Deutschen Gesellschaft für Gastroenterologie, Verdauungs‐ und Stoffwechselkrankheiten (DGVS) zur Prophylaxe, Diagnostik und Therapie der Hepatitis‐B‐Virusinfektion.^1^

Das Kapitel wurde gemeinsam entwickelt mit Prof. Dr. Markus Cornberg, Medizinische Hochschule Hannover, der von der Deutschen Gesellschaft für Gastroenterologie, Verdauungs‐ und Stoffwechselkrankheiten (DGVS) nominiert wurde.

**Empfehlungen**:

**Table:** 

3.8‐1 | modifiziert [2025] Ein Screening auf Hepatitis A, D oder E als Routinemaßnahme vor Beginn einer systemischen Behandlung durchzuführen, *wird nicht empfohlen*.	**↓↓**	starker konsens Konsensbasiert Gemeinsam mit der DGVS entwickelt

a. **Screening**

Die Untersuchung auf Hepatitis A, D und E sollte nur dann durchgeführt werden, wenn dies durch die Anamnese, erhöhte Leberenzyme, klinische Anzeichen und Symptome indiziert ist, jedoch nicht als routinemäßige Screening‐Parameter.

**Table:** 

3.8‐2 | modifiziert [2025] Wir *empfehlen*, die Patienten routinemäßig auf Hepatitis B (HBsAg, anti‐HBs, anti‐HBc) zu untersuchen, bevor eine Behandlung mit Ciclosporin, Deucravacitinib, Methotrexat oder Biologika begonnen wird.	**↑↑**	konsens Konsensbasiert Gemeinsam mit der DGVS entwickelt
3.8‐3 | neu [2025] Bei der Konstellation anti‐HBc positiv und HBsAg negativ *empfehlen* wir eine okkulte HBV‐Infektion mittels HBV‐DNA‐Bestimmung auszuschließen.	**↑↑**	starker konsens Konsensbasiert Gemeinsam mit der DGVS entwickelt
3.8‐4 | modifiziert [2025] Wir *empfehlen*, bei der Beurteilung der Hepatitis‐B‐Testergebnisse den in Abbildung 1 dargestellten Algorithmus zu befolgen.	**↑↑**	starker konsens Konsensbasiert Gemeinsam mit der DGVS entwickelt
3.8‐5 | modifiziert [2025] Es *kann empfohlen werden*, Patienten routinemäßig auf Hepatitis C zu untersuchen, bevor eine Behandlung mit Deucravacitinib, Methotrexat oder Biologika begonnen wird.	**↑**	starker konsens ^1^ Konsensbasiert Gemeinsam mit der DGVS entwickelt
3.8‐6 | modifiziert [2025] Bei positiven Befunden für Hepatitis‐C‐Antikörper *wird* eine Testung auf Hepatitis‐C‐RNA *empfohlen*.	**↑↑**	starker konsens Konsensbasiert Gemeinsam mit der DGVS entwickelt
3.8‐7 | modifiziert [2025] Bei positiven Befunden für Hepatitis‐C‐Antikörper und Hepatitis‐C‐RNA *wird* eine Überweisung an eine/n Facharzt/‐ärztin mit Erfahrung in der Behandlung von Virushepatitis *empfohlen*.	**↑↑**	starker konsens Konsensbasiert Gemeinsam mit der DGVS entwickelt

^1^ Fünf Enthaltungen aufgrund von Interessenkonflikten.

**ABBILDUNG 1:**
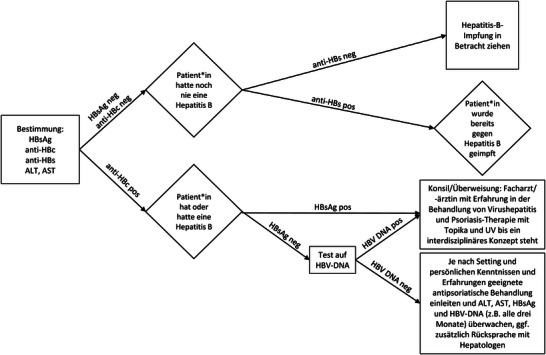
Algorithmus für die Interpretation der Hepatitis‐B‐Testergebnisse.

Wenn vor einer immunsuppressiven Therapie der Ausschluss einer Virushepatitis einmalig erfolgt ist und keine anamnestischen/laborchemischen Hinweise auf eine Infektion vorliegen, kann bei Therapieumstellungen auf eine erneute Testung verzichtet werden.

Wenn nach einer kürzlich erfolgten HCV‐Behandlung die HCV‐Antikörper positiv sind und der Test auf HCV‐RNA negativ ist, sollte eine Mitbeurteilung bezüglich einer Leberfibrose durch einen Hepatologen/Experten für Erkrankungen der Leber erfolgen.

Bei positiven Anti‐HCV‐Antikörpern und negativer HCV‐RNA ohne vorherige Anti‐HCV‐Behandlung handelt es sich um eine ausgeheilte HCV‐Infektion. Diese erfordert keine weitere Überweisung an eine/n Facharzt/‐ärztin mit Erfahrung in der Behandlung von Virushepatitis.

b. **Behandlung**

**Table:** 

3.8‐8 | modifiziert [2025] ***H** **BsAg** **pos**. **oder** **HBV** **DNA** **pos***. *Wir empfehlen*, dass die Therapieauswahl bei Patienten mit einem positiven Testergebnis für HBsAg oder positiver HBV‐DNA zusammen mit einem Facharzt/‐ärztin mit Erfahrung in der Behandlung von Virushepatitis getroffen wird.^*^ (^*^Zur Indikation einer antiviralen Therapie mit Nukleos(t)idanalogon zur Prophylaxe der Reaktivierung unter immunsuppressiver Therapie siehe: S3‐Leitlinie der Deutschen Gesellschaft für Gastroenterologie, Verdauungs‐ und Stoffwechselkrankheiten (DGVS) zur Prophylaxe, Diagnostik und Therapie der Hepatitis‐B‐Virusinfektion) ^1^	**↑↑**	starker konsens Konsensbasiert Gemeinsam mit der DGVS entwickelt
3.8‐9 | neu [2025] ***anti‐HBc‐positiv** **, HBV‐DNA negativ** **und HBsAg‐negativ*** ***anti‐HCV‐positiv und HCV‐RNA‐negativ*** Die identifizierte Evidenz ermöglicht keine spezifische Empfehlung einer der in der Leitlinie behandelten Psoriasistherapien bei Patienten mit Virushepatitis folgender Serumkonstellation (Hepatitis B: anti‐HBc‐positiv/HBsAg‐negativ/HBV‐DNA negativ oder Hepatitis C: anti‐HCV‐positiv/HCV‐RNA‐negativ). Für diese Patienten *kann empfohlen werden*, die Therapieoption aus der Leitlinie auszuwählen, die am besten für die Psoriasiserkrankung der Patienten geeignet ist, wobei zu bedenken ist, dass für die neueren Medikamente die Datenlage zum Reaktivierungsrisiko noch sehr begrenzt ist.	**↑**	starker konsens ^1^ Evidenz‐ und konsensbasiert (siehe Evidenzbericht, Kapitel 3) Gemeinsam mit der DGVS entwickelt LoE: 3 (OECBM)

1 Fünf Enthaltungen aufgrund von Interessenkonflikten.

Die Daten, die im Rahmen der systematischen Evidenzrecherche für diese Leitlinie identifiziert wurden, reichen nicht aus, um Empfehlungen für oder gegen die Verwendung der vorhandenen antipsoriatischen Medikamente bei Patienten mit einer mittelschweren bis schweren Psoriasis und einer Hepatitis B zu geben.

Tabelle 13 im Evidenzbericht bietet eine Zusammenfassung der berichteten Fälle von Reaktivierungen. Die gemeldeten Fälle müssen in Korrelation zum Zulassungsdatum gesehen werden, insbesondere mit den Jahren und der Anzahl der Patienten mit Psoriasis und Virushepatitis, die dem jeweiligen Medikament ausgesetzt waren. Dies gilt aktuell insbesondere für Deucravacitinib, wo die Anzahl der exponierten Patienten noch gering ist. Detaillierte Informationen dazu finden Sie im Leitlinienreport und Evidenzbericht. Die S3‐Leitlinie der Deutschen Gesellschaft für Gastroenterologie, Verdauungs‐ und Stoffwechselkrankheiten (DGVS) zur Prophylaxe, Diagnostik und Therapie der Hepatitis‐B‐Virusinfektion bewertet das Risiko für eine HBV‐Reaktivierung bei Patienten mit HBsAg neg./anti‐HBc pos. unter MTX und TNFi als „niedrig“, unter Ustekinumab als „moderat“. Zu den anderen Therapieoptionen findet sich dort noch keine Einschätzung. Für diese Patientengruppe sollen entsprechend der genannten Leitlinie bei Immunsuppression mit „moderatem oder niedrigem Reaktivierungsrisiko engmaschige Kontrollen“ (siehe unten) erfolgen, oder „in Sonderfällen, zum Beispiel sehr langfristige Immunsuppression, unzureichende Adhärenz zur engmaschigen Kontrolle oder ungünstige Risikofaktoren (Alter, Tumorentität, begleitende Lebererkrankung o. Ä.) kann eine prophylaktische antivirale Therapie durchgeführt werden“.^1^

Für einige der Therapieoptionen wird in den Fachinformationen eine Virushepatitis als Kontraindikation genannt, obwohl die klinische Praxis, verfügbare Fallserien oder Registerdaten auf ein Sicherheitsprofil hinweisen, welche mit den Therapien vergleichbar ist, bei denen dies nicht als Kontraindikation erwähnt wird. Dies gilt insbesondere für Methotrexat, wobei die Studiendaten zumindest keine Zunahme der Leberfibrose anzeigen.^2^

c. **Überwachung auf Reaktivierungen während der Behandlung**

**Table:** 

3.8‐10 | neu [2025] Zur Überwachung der Reaktivierung einer viralen Hepatitis bei Patienten, die Anti‐HBc‐positiv/HBsAg‐negativ sind, *wird empfohlen*, regelmäßig ALT, AST, HBsAg und HBV‐DNA zu überprüfen (mindestens alle drei Monate).	**↑↑**	starker konsens Konsensbasiert Leitlinien‐adaptierung DGVS
3.8‐11 | geprüft [2025] Wir *empfehlen*, alle Behandlungseinleitungen und Folgebesuche von Patienten mit Psoriasis und konkomittierender Hepatitis B oder C an entsprechende Register zu melden.	**↑↑**	starker konsens Konsensbasiert Gemeinsam mit der DGVS entwickelt

Bei der Empfehlung 3.8‐10 handelt es sich um eine Adaptierung einer im Hintergrundtext befindlichen Aussage aus der S3‐Leitlinie zur Prophylaxe, Diagnostik und Therapie der Hepatitis‐B‐Virusinfektion der Deutschen Gesellschaft für Gastroenterologie, Verdauungs‐ und Stoffwechselkrankheiten (DGVS)^1^ (AWMF‐Registernummer 021‐011).

Die Aussage zu dem Zeitabstand der Kontrolluntersuchungen wurde in Anlehnung an die üblichen Abstände der Arztkontakte der adressierten Patientengruppe angepasst, lässt kürzere Kontrollintervalle jedoch ausdrücklich zu.

## TUBERKULOSE: WIE SOLL VOR UND WÄHREND EINER SYSTEMTHERAPIE AUF TUBERKULOSE GESCREENT WERDEN?

Dieses Kapitel basiert auf den Vorversionen der Leitlinie.^3^, ^4^, ^5^, ^6^ Die Einzelheiten der Recherche sind im Evidenzbericht im Kapitel 4 dokumentiert.

Das Kapitel wurde in Zusammenarbeit mit der Deutschen Gesellschaft für Pneumologie und Beatmungsmedizin (DGP) durch das deutsche Zentralkomitee zur Bekämpfung der Tuberkulose (DZK) vollständig überarbeitet.

Eine Tuberkulose (TB) kann unerkannt und unbehandelt ein lebensbedrohliches Krankheitsbild darstellen. Während TB weltweit mit über 10 Millionen Neuerkrankungen pro Jahr eine der häufigsten Infektionserkrankungen darstellt, ist die Erkrankung in Deutschland mit 4000 bis 5000 Neuerkrankungen pro Jahr selten.^7^, ^8^

Wie hoch allerdings der Anteil der Personen mit einer latenten TB‐Infektion weltweit und in Deutschland ist, kann nur geschätzt werden.^9^ Dabei erkranken nur 5% bis 10% aller Infizierten im Laufe ihres Lebens. Dieses Risiko wird durch Erkrankungen des Immunsystems wie auch immunsupprimierende Medikamente erhöht.

Nach Einführung der TNF‐Inhibitoren (TNFi) kam es bei damals noch nicht vorgeschriebenem TB‐Screening zum gehäuften Auftreten von komplexen und schwer verlaufenden Tuberkulose‐Erkrankungen, die zur Einführung eines Screenings vor diesen Therapien führte. Für alle danach entwickelten immunmodulatorischen Therapien wurden Personen mit latenter TB‐Infektion in den Zulassungsstudien ausgeschlossen oder präventiv antituberkulös behandelt.^10^

Einschätzungen des Reaktivierungsrisikos basieren daher hauptsächlich auf Überlegungen zu Wirkmechanismen, dem Fehlen von gemeldeten Tuberkulosefällen unter immunmodulatorischen Therapien, internationalen Einschätzungen^11^, ^12^ sowie Risiko‐Nutzen‐Abwägungen. Aufmerksamkeit bezüglich möglicher Symptome einer Tuberkulose bleibt weiterhin geboten, um eventuell auftretende Tuberkulosefälle rechtzeitig zu erkennen, zu behandeln und zu melden.

In diesem Kapitel wird das Screening auf eine latente TB‐Infektion und im darauffolgenden Kapitel das weitere Vorgehen beim Vorliegen eines positiven Interferon‐Gamma‐Release Assays (IGRA) dargestellt.

**Table:** 

3.9‐1 | neu [2025] Vor Einleitung einer Therapie mit einem Biologikum oder mit Deucravacitinib** *wird* die Durchführung eines Interferon‐Gamma‐Release‐Assays (IGRA) zum Ausschluss einer latenten TB‐Infektion *empfohlen*. **Deucravacitinib: Aufgrund mangelnder Daten zum Reaktivierungsrisiko	**↑↑**	starker konsens ^1^ Konsensbasiert Gemeinsam mit der DGP entwickelt
3.9‐2 | neu [2025] Zum Ausschluss einer Tuberkulose *wird* die zusätzliche Durchführung eines Röntgen des Thorax bei einem negativen IGRA nur *empfohlen*, wenn: ‐ eine erhöhte Wahrscheinlichkeit für das Vorliegen einer Tuberkulose besteht (siehe Hintergrundtext) oder ‐ Faktoren vorliegen, die das Risiko eines falsch negativen IGRA Tests erhöhen (siehe Hintergrundtext).	**↑↑**	starker konsens Konsensbasiert Gemeinsam mit der DGP entwickelt

^1^ Drei Enthaltungen aufgrund von Interessenkonflikten.

*Faktoren, die das Risiko eines falsch negativen IGRA erhöhen*:

‐ Immunsuppression (iatrogen und/oder erkrankungsbedingt),^13^
‐ Lymphozytopenie,^14^
‐ Vorangegangene Impfung mit einem Lebendimpfstoff innerhalb der letzten 4–6 Wochen,^15^
‐ Fulminante TB‐Erkrankung, zum Beispiel Miliartuberkulose.^16^

Bis zur Version 2021 der deutschen Psoriasisleitlinie^6^ wurde auch vor Einleitung von MTX ein Screening auf eine latente TB‐Infektion empfohlen. Der Grund war damals nicht primär die Sorge vor Reaktivierungen einer latenten TB‐Infektion unter MTX. Es war als Vorsichtsmaßnahme für Situationen zu verstehen, in denen im weiteren Krankheitsverlauf eine Umstellung von MTX auf ein Biologikum wahrscheinlich erschien. Dies erfolgte in Abwägung, dass die Testgenauigkeit eines IGRA während einer Therapie mit MTX verringert sein könnte. Dies kann weiterhin abgewogen werden.

**Table:** 

3.9‐3 | neu [2025] Eine Wiederholung des IGRAs nur aufgrund fester Zeitintervalle oder nur aufgrund des Wechsels von einem Medikament auf ein anderes, ohne ein relevantes Expositionsrisiko *wird nicht empfohlen*.	**↓↓**	starker konsens Konsensbasiert Gemeinsam mit der DGP entwickelt
3.9‐4 | neu [2025] Eine Wiederholung des IGRA im Verlauf *wird* bei einem erhöhten Risiko einer TB‐Exposition *empfohlen* (siehe Hintergrundtext: Anhaltspunkte für eine erhöhte Wahrscheinlichkeit für ein Expositionsrisiko).	**↑↑**	starker konsens Konsensbasiert Gemeinsam mit der DGP entwickelt

*Anhaltspunkte für eine erhöhte Wahrscheinlichkeit einer TB‐Exposition^15^/Vorliegen einer TB*:

‐ Direkter Kontakt mit einer Person mit aktiver TB,
‐ Herkunft oder längerer Aufenthalt (> 3 Monate) in einem Hochprävalenzland für Tuberkulose (> 100 Neuerkrankungen/100 000 Einwohner),
‐ Aufenthalt oder Tätigkeiten in einer Unterbringung für Migranten, Asylsuchende, Wohnungslose oder im Vollzug,
‐ Tätigkeit in einer medizinischen Einrichtung für TB‐Erkrankte.
‐ Für weitere Informationen verweist die Leitliniengruppe auf die S2k‐Leitlinie „Tuberkulose im Erwachsenenalter“^17^ (AWMF‐Registernummer: 020‐019) sowie auf die Informationen des Robert Koch‐Institutes (RKI; https://www.rki.de/DE/Content/InfAZ/T/Tuberkulose/Tuberkulose.html) und des Deutschen Zentralkomitees zur Bekämpfung der Tuberkulose (DZK; https://www.dzk‐tuberkulose.de/).

## TUBERKULOSE: WIE SOLL DAS MANAGEMENT BEI PATIENTEN MIT PSORIASIS UND EINEM POSITIVEM INTERFERON GAMMA RELEASE ASSAY (IGRA) ERFOLGEN?

Dieses Kapitel basiert auf den Vorversionen der Leitlinie.^3^, ^4^, ^5^, ^6^ Die Einzelheiten der Recherche sind im Evidenzbericht im Kapitel 4 dokumentiert.

Dieses Kapitel wurde in Zusammenarbeit mit der Deutschen Gesellschaft für Pneumologie und Beatmungsmedizin (DGP) durch das deutsche Zentralkomitee zur Bekämpfung der Tuberkulose (DZK) vollständig überarbeitet.

### Vorgehen bei positivem Befund im IGRA

**Table:** 

3.10‐1 | neu [2025] Bei einem positiven IGRA *wird* zur Unterscheidung zwischen aktiver TB und latenter TB‐Infektion folgendes Vorgehen *empfohlen*: ‐ Anamnese: Expositionsrisiko, Anzeichen und Symptome einer aktiven TB (zum Beispiel Husten, Hämoptysen, Fieber, Gewichtsverlust, Nachtschweiß), ‐ Körperliche Untersuchung (unter anderem Palpation der Lymphknoten), ‐ Röntgen des Thorax in p.a., wenn notwendig in zwei Ebenen.	**↑↑**	starker konsens Konsensbasiert Gemeinsam mit der DGP entwickelt
3.10‐2 | neu [2025] Bei auffälliger Klinik oder unklarem Röntgenbefund beziehungsweise bei Hinweisen auf eine TB im Röntgenbefund *wird* eine Überweisung zu einem Facharzt mit Erfahrung in der Behandlung von Tuberkulose *empfohlen*.	**↑↑**	starker konsens Konsensbasiert Gemeinsam mit der DGP entwickelt

*Zur Diagnostik der Tuberkulose verweist die Leitliniengruppe auf die S2k‐Leitlinie „Tuberkulose im Erwachsenenalter“* *17* *(AWMF‐Registernummer: 020‐019)*.

### Vorbemerkung zur Therapieauswahl

Bei Vorliegen einer Tuberkulose ist die Zusammenarbeit mit Pneumologen/Infektiologen unter anderem wichtig, um die Notwendigkeit eine Pausierung oder Umstellung der antipsoriatischen Therapie zu diskutieren.^17^

Das nachfolgende Kapitel befasst sich mit dem Vorgehen für die weitere Behandlung der Psoriasis vulgaris bei Vorliegen einer latenten TB‐Infektion sowie einer potenziellen präventiven Therapie. Die Empfehlungen berücksichtigen auch eine Schaden‐Nutzen‐Abwägung der präventiven antituberkulösen Therapie. Mögliche unerwünschte Arzneimittelwirkungen sind in der S2k‐Leitlinie “Tuberkulose im Erwachsenenalter“ ^17^ (AWMF‐Registernummer: 020‐019) aufgeführt.

### Auswahl der geeigneten antipsoriatischen Therapie und Indikationen zur präventiven antituberkulösen Therapie

**Table:** 

3.10‐3 | modifiziert [2025] Für Patienten mit latenter TB‐Infektion *wird* die Auswahl einer der folgenden Optionen *empfohlen*: ‐ Acitretin, Apremilast, Ciclosporin, Dimethylfumarat, MTX oder Fototherapie.	**↑↑**	konsens ^1^ Konsensbasiert Gemeinsam mit der DGP entwickelt
3.10‐4 | neu [2025] Eine präventive antituberkulöse Therapie** aufgrund der Therapieeinleitung mit Acitretin, Apremilast, Ciclosporin, Dimethylfumarat oder MTX *kann nicht empfohlen werden*. **Wenn keine anderen Indikationen zur Einleitung einer präventiven antituberkulösen Therapie bestehen (siehe S2‐k‐Leitlinie „Tuberkulose im Erwachsenenalter“^17^)	**↓**	konsens ^2^ Konsensbasiert Gemeinsam mit der DGP entwickelt

^1^ Eine Enthaltung aufgrund von Interessenkonflikten

^2^ zwei Enthaltungen aufgrund von Interessenkonflikten.

Eine dokumentierte Recherche nach systematischen Reviews (SR) in der Datenbank Medline via Ovid identifizierte keine hochwertigen SR zu der Fragestellung des Reaktivierungsrisikos unter Anwendung von Acitretin, Ciclosporin, Fumaraten oder MTX.

Der Einsatz dieser Medikamente erfolgt seit Jahrzehnten (Ausnahme: Apremilast seit 2015). In der deutschen Psoriasisleitlinie wurde für die Wirkstoffe Acitretin, Apremilast, Ciclosporin sowie Dimethylfumarat bisher kein Screening auf eine latente TB‐Infektion beziehungsweise keine präventive antituberkulöse Therapie empfohlen.^6^ Auch in den Fachinformationen wird kein Screening auf eine latente TB‐Infektion oder Tuberkulose empfohlen. Daten aus der Versorgungsforschung weisen darauf hin, dass dies auch mehrheitlich so praktiziert wird.^18^ Aus diesem Vorgehen resultierende Sicherheitssignale sind der Leitliniengruppe weder aus ihrer klinischen Erfahrung noch aus der ihr bekannten Literatur bekannt.

Für MTX zeigte sich in den Daten der Versorgungsforschung dagegen ein deutlich heterogener Umgang bezüglich einer präventiven antituberkulösen Therapie bei Vorliegen einer latenten TB‐Infektion.^18^

Der Wirkstoff MTX wird seit den 50er Jahren für eine Vielzahl von hämatoonkologischen, rheumatologischen, immunologischen und dermatologischen Erkrankungen eingesetzt. In den Fachinformationen von MTX wurde in der Regel kein explizites Tuberkulosescreening beziehungsweise keine präventive antituberkulöse Therapie gefordert.

Es ist daher anzunehmen, dass eine Vielzahl von Patienten weltweit mit diesem Medikament behandelt wurden, bei denen eine latente TB‐Infektion vorlag. Gehäufte schwere oder atypische Verläufe von Reaktivierungen, wie diese unter TNFi ^19^ berichtet wurden, sind der Leitliniengruppe nicht bekannt.

Ein aktueller ausreichend hochwertiger systematischer Review zur Beurteilung des Reaktivierungsrisikos einer latenten TB‐Infektion unter MTX konnte nicht identifiziert werden. Mittels Handsuche wurden zwei populationsbasierte Fall‐Kontroll‐Studien identifiziert.^20^, ^21^ Eine schätzte das Reaktivierungsrisiko unter MTX bei Patienten mit rheumatoider Arthritis als gering ein.^21^ Eine weitere sieht keine Assoziation zwischen MTX und einem erhöhten Risiko für eine aktive Tuberkulose bei Patienten mit rheumatoider Arthritis im Alter ≥ 67 Jahren.^20^

Die identifizierte Evidenz wird als nicht ausreichend eingeschätzt, um ein relevantes Reaktivierungsrisiko beziehungsweise ein Risiko für besonders schwere oder atypische Verläufe festzustellen und um eine Empfehlung für eine präventive antituberkulöse Therapie zu rechtfertigen.

Gegen eine präventive antituberkulöse Therapie bei latenter TB‐Infektion bei MTX sprechen unter anderem der mögliche Schaden der präventiven antituberkulösen Therapie, mögliche Arzneimittelinteraktionen sowie die mit hohem Aufwand verbundene mehrmonatige Einnahme.

Die Leitliniengruppe hat sich in Abwägung der oben genannten Aspekte und unter Berücksichtigung ihrer klinischen Erfahrungen gegen eine Empfehlung zur präventiven antituberkulösen Therapie bei den in Empfehlung 3.10‐4 genannten Wirkstoffen entschieden, es sei denn, es liegen unten genannte Indikationen vor (siehe Absatz: „Allgemeine Indikation zur präventiven antituberkulösen Therapie bei Patienten mit latenter TB‐Infektion“).

**Table:** 

3.10‐5 | modifiziert [2025] Für Patienten mit latenter TB‐Infektion *kann* die Auswahl einer der folgenden Optionen *empfohlen werden*: ‐ IL17i, ‐ IL23i oder ‐ IL12/23p40i.	**↑**	starker konsens ^1^ Evidenz‐ und konsensbasiert (Siehe Evidenzbericht Kapitel 4) LoE: 3‐4 (OCEBM) Gemeinsam mit der DGP entwickelt
3.10‐6 | neu [2025] Eine präventive antituberkulöse Therapie aufgrund einer Therapieeinleitung mit IL12/23p40i** *kann empfohlen werden*. **Abweichung zur Fachinformation, lt. Fachinformation „muss“ bei Ustekinumab eine präventive antituberkulöse Therapie eingeleitet werden. Die Leitliniengruppe konnte keine Daten identifizieren, die diese Unterscheidung ausreichend begründet. Eine Aufklärung und Einbindung des Aspektes in die gemeinsame Entscheidungsfindung sind erforderlich.	**↑**	starker konsens ^1^ Konsensbasiert Gemeinsam mit der DGP entwickelt
3.10‐7 | neu [2025] Eine präventive antituberkulöse Therapie aufgrund einer Therapieeinleitung mit IL17i oder IL23i *kann erwogen werden*.	**0**	starker konsens ^1^ Konsensbasiert Gemeinsam mit der DGP entwickelt

^1^ Fünf Enthaltungen aufgrund von Interessenkonflikten.

Das Generieren von Daten über das Reaktivierungsrisiko neuerer Wirkstoffe ist nicht oder nur begrenzt möglich, da Patienten mit latenter TB‐Infektion bereits in den Zulassungsstudien ausgeschlossen oder präventiv antituberkulös behandelt wurden (siehe auch Kapitel TB‐Screening).

Grundlage für die Empfehlung 3.10‐5 bildet ein in einer systematischen Recherche identifizierter systematischer Review (SR), der das Reaktivierungsrisiko einer latenten TB‐Infektion bei Patienten mit Psoriasis und Therapie mit Biologika mit und ohne präventive Therapie untersuchte.^22^

Da die Autoren des SR^22^ bei ihrer Evidenzsynthese nicht differenzierten, ob die Reaktivierungen unter präventiver antituberkulöser Therapie stattfanden oder nicht, entschied sich die Leitliniengruppe, diese Informationen aus den in dem SR eingeschlossenen Primärstudien nachzuextrahieren (Details siehe Evidenzbericht, Kapitel 4).

Aus Sicht der Leitliniengruppe ergaben sich daraus keine ausreichenden Hinweise auf ein Reaktivierungsrisiko unter IL17‐ oder IL23‐Inhibitoren. Für diese Medikamentengruppen sieht die Fachinformation die Einleitung einer präventiven antituberkulösen Therapie als fakultativ an. Auch andere internationale Experten schätzen für diese Medikamentengruppe das Reaktivierungsrisiko geringer ein.^11^, ^12^

Bei Einleitung des IL12/23p40 Inhibitor Ustekinumab fordert die Fachinformation die Durchführung einer präventiven antituberkulösen Therapie im Falle einer latenten TB‐Infektion. Der Hersteller konnte nach Anfrage keine Daten zur Verfügung stellen, die spezifisch ein erhöhtes Risiko einer Reaktivierung belegen. Die Leitliniengruppe erkennt in den identifizierten Daten^22^ keine Hinweise für ein erhöhtes Reaktivierungsrisiko, wenngleich die untersuchten Fallzahlen sehr gering waren (siehe Evidenzbericht, Kapitel 4).

**Table:** 

3.10‐8 | neu [2025] Bei Patienten mit latenter TB‐Infektion *kann* die Anwendung von Deucravacitinib** *nicht empfohlen werden*, es sei denn, es gibt keine anderen geeigneten Behandlungsmöglichkeiten. **Aufgrund mangelnder Daten zum Reaktivierungsrisiko	**↓**	starker konsens ^1^ Konsensbasiert Gemeinsam mit der DGP entwickelt
3.10‐9 | neu [2025] Eine präventive antituberkulöse Therapie aufgrund einer Therapieeinleitung mit Deucravacitinib** *kann empfohlen werden*. **Aufgrund mangelnder Daten zum Reaktivierungsrisiko	**↑**	konsens ^1^ Konsensbasiert Gemeinsam mit der DGP entwickelt

^1^ Zwei Enthaltungen aufgrund von Interessenkonflikten.

Die Daten zum Reaktivierungsrisiko einer latenten TB‐Infektion unter Therapie mit Deucravacitinib sind noch unzureichend für eine Bewertung. Die Fachinformation empfiehlt die Einleitung einer präventiven antituberkulösen Therapie. Aufgrund fehlender anderweitiger Erfahrungen und einem unbekannten Reaktivierungsrisiko schließt sich die Leitliniengruppe dieser Empfehlung an.

**Table:** 

3.10‐10 | geprüft [2025] Bei Patienten mit latenter TB‐Infektion *wird* die Anwendung von TNFi *nicht empfohlen*, es sei denn, es gibt keine anderen geeigneten Behandlungsmöglichkeiten.	**↓↓**	starker konsens ^1^ Konsensbasiert Gemeinsam mit der DGP geprüft
3.10‐11 | neu [2025] Eine präventive antituberkulöse Therapie aufgrund einer Therapieeinleitung mit TNFi *wird empfohlen*.	**↑↑**	konsens ^1^ Konsensbasiert Gemeinsam mit der DGP entwickelt

^1^ Zwei Enthaltungen aufgrund von Interessenkonflikten.

Ein in einer Handsuche identifizierter systematischer Review zeigt, dass das Risiko für eine aktive Tuberkulose unter TNFi im Vergleich zur Kontrollgruppe (nicht‐TNFi oder Placebo) erhöht ist (46/7912 versus 3/3967; OR 1,94 (95%‐Konfidenzintervall [KI] 1,1; 3,44), I^2^ = 0%, N = 29 RCTs) ^23^. Extrapulmonale und disseminierte Manifestationen wurden unter TNFi vermehrt berichtet.^19^

### Allgemeine Indikation zur präventiven antituberkulösen Therapie bei Patienten mit latenter TB‐Infektion

Für die Prüfung der Indikation zu einer präventiven antituberkulösen Therapie bei einer latenten TB‐Infektion ‐ auch unabhängig von der Einleitung einer Psoriasistherapie – verweist die Leitliniengruppe auf die S2k‐Leitlinie “Tuberkulose im Erwachsenenalter“^17^ (AWMF‐Registernummer: 020‐019).

Indikationen zur präventiven antituberkulösen Therapie einer latente TB‐Infektion bestehen insbesondere bei (adaptiert nach Schaberg et al.^17^):

‐ Kontaktpersonen zu Personen mit aktiver TB,
‐ Menschen mit unzureichender Kontrolle einer HIV‐Erkrankung,
‐ Menschen vor TNFi‐Therapie und gegebenenfalls anderen Biologika/JAK‐Inhibitoren,
‐ Menschen mit schweren Grunderkrankungen, die eine Immunsuppression bedingen,
‐ Menschen vor geplanter beziehungsweise nach durchgeführter Organ‐ oder hämatologischer Transplantation,
‐ Menschen aus TB‐Hochprävalenzländern.

### Form der präventiven antituberkulöse Therapie

**Table:** 

3.10‐12 | neu [2025] Als präventive antituberkulöse Therapie *wird* entweder die Gabe von Rifampicin (4 Monate) oder Isoniazid + Rifampicin (3 Monate) oder Isoniazid (9 Monate) *empfohlen*.	**↑↑**	starker konsens Konsensbasiert Gemeinsam mit der DGP entwickelt

Die Empfehlungen zur Art und Dauer der präventiven antituberkulösen Therapie orientieren sich an der S2k‐Leitlinie “Tuberkulose im Erwachsenenalter“^17^ (AWMF‐Registernummer: 020‐019). Auf diese verweist die Leitliniengruppe auch für weitere Maßnahmen, die vor, während und nach der präventiven antituberkulösen Therapie zu beachten sind. Die kürzeren Rifampicin‐haltigen Therapieregime werden auf Grund der besser sicherzustellenden Adhärenz in der Regel bevorzugt. Auf Interaktionen und gegebenenfalls Therapieanpassungen anderer Medikamente ist bei Einsatz von Rifampicin zu achten.

**Table:** 

3.10‐13 | neu [2025] Zwischen dem Beginn der präventiven antituberkulösen Therapie und der Einleitung der immunsuppressiven Therapie kann in der Regel ein Abstand von 4 Wochen empfohlen werden.	**↑**	starker konsens Konsensbasiert Gemeinsam mit der DGP entwickelt

Der üblicherweise empfohlene Zeitabstand von 4 Wochen zwischen Einleitung einer präventiven antituberkulösen Therapie und der immunsuppressiven Therapie basiert nicht auf Studiendaten, sondern auf theoretischen Abwägungen. Die Leitliniengruppe spricht bewusst eine abgeschwächte Empfehlung aus, und weist darauf hin, dass bei dringend behandlungsbedürftiger Psoriasis auch kürzere Zeiträume zwischen dem Beginn der präventiven antituberkulösen Therapie und der Einleitung der antipsoriatischen Systemtherapie für möglich erachtet werden.

### Gemeinsame Entscheidungsfindung

Die Einleitung einer präventiven antituberkulösen Therapie erfordert eine Risiko‐Nutzen‐Abwägung gemeinsam mit den Patienten (Tabelle 4). Dies ist in der Leitlinie zur Therapie der Tuberkulose ausführlich dargestellt.^17^ Eine Aufklärung über tuberkulosespezifische Symptome sowie das verbleibende Risiko einer erneuten Ansteckung/Erkrankung auch nach erfolgter präventiver antituberkulöser Therapie ist durchzuführen.

**TABELLE 4:** Potenzielle Gründe für beziehungsweise gegen eine präventive antituberkulöse Therapie.

Gründe für eine präventive antituberkulöse Therapie	Gründe gegen eine präventive antituberkulöse Therapie
Jüngeres Alter, generelle Vermeidung einer Reaktivierung bei erhöhtem Lebenszeitrisiko einer TB‐Erkrankung (Siehe S2k‐Leitlinie „Tuberkulose im Erwachsenenalter“^17^)	Erhöhtes Risiko einer Hepatotoxizität (zum Beispiel Alter > 60 Jahren (v.a. relevant für Therapie mit INH), bekannte Lebererkrankung)
Sonstige vorliegende Immunsuppression	Umfangreiche Komedikation, Gefahr von kumulativen UAWs, Arzneimittelinteraktionen
geplante Therapie mit höherem Reaktivierungsrisiko	geplante Therapie mit geringem Reaktivierungsrisiko
Relevanter Kontakt zu einem Indexfall bei positivem IGRA und negativem Befund im Röntgen des Thorax	Adhärenz nicht sicherzustellen

Basierend auf einer von den Autoren eingebrachten Netzwerkmetaanalyse^24^ sowie auf den Aussagen nationaler und internationaler Leitlinien^17^, ^25^ sieht die Leitliniengruppe die präventive antituberkulöse Therapie als wirksam an, wobei sich die Ergebnisse je nach Therapieauswahl und ‐Dauer unterscheiden.

### Förderung der Versorgungsforschung

**Table:** 

3.10‐14 | neu [2025] Es *wird empfohlen*, Patienten mit latenter TB‐Infektion, bei denen eine Therapie mit IL17i, IL23i oder IL12/23p40i eingeleitet wurde, in Register/wissenschaftlichen Studien zu erfassen.	**↑↑**	starker konsens Konsensbasiert Gemeinsam mit der DGP entwickelt

### Management der aktiven Tuberkulose

**Table:** 

3.10‐15 | neu [2025] Beim Vorliegen einer aktiven TB *wird* die Entwicklung eines Therapiekonzeptes für die Psoriasis und die TB gemeinsam mit einer/m Facharzt/‐ärztin mit Erfahrung in der Behandlung von Tuberkulose *empfohlen*.	**↑↑**	starker konsens Konsensbasiert Gemeinsam mit der DGP entwickelt

*Die Leitliniengruppe verweist hierzu auch auf die S2k‐Leitlinie “Tuberkulose im Erwachsenenalter“* ^*17*^ *(AWMF‐Registernummer: 020‐019)*.

*Für die Kapitel „Kinderwunsch/Schwangerschaft“, „Psoriasisarthritis“ sowie „Immunogenität: Entwicklung von Antikörpern gegen die zielgerichteten Therapien bei Psoriasis“ siehe Langfassung*.

## IMPFUNGEN: WIE SOLLTEN IMPFUNGEN BEI PATIENTEN MIT PSORIASIS UNTER SYSTEMISCHER BEHANDLUNG GEHANDHABT WERDEN?

Im Februar 2023 wurde ein nicht systematischer Literatur‐Review durchgeführt.

**Ergebnisse/Empfehlungen**:

Eine Psoriasiserkrankung wird von der Leitliniengruppe nicht per se als Grund angesehen, von den Standard‐Impfempfehlungen/nationalen Impfrichtlinien abzuweichen.

Für Patienten, die eine systemische immunmodifizierende Therapie bei Psoriasis beginnen:

‐ Der optimale Zeitpunkt für Impfungen liegt vor Beginn einer systemischen immunsuppressiven Therapie.
‐ Überprüfen Sie den Impfstatus und führen Sie entsprechende Impfungen gemäß den nationalen Impfrichtlinien durch, bevor Sie mit einer systemischen immunsuppressiven Therapie beginnen, sofern möglich.
‐ Überprüfen Sie die produktspezifischen Fachinformationen zur empfohlenen Zeitspanne für den Beginn einer systemischen immunsuppressiven Medikation nach der Impfung.

Für Patienten, die eine systemische immunsuppressive Therapie bei Psoriasis erhalten:

‐ Die Immunantwort auf Impfungen wird von mehreren Faktoren beeinflusst, darunter die Art und Dosis der systemischen immunsuppressiven Therapie, die Art des Impfstoffs (Lebend‐ oder Totimpfstoff), intrinsische Faktoren (zum Beispiel Alter, Begleiterkrankungen) sowie extrinsische Faktoren (zum Beispiel vorbestehende Immunität durch vorherige Antigenexposition).^26^
‐ Überprüfen der nationalen Impfrichtlinien auf Impfanforderungen während der Therapie.
‐ Überprüfen der Fachinformation hinsichtlich des empfohlenen zeitlichen Abstandes für die Einnahme einer systemischen immunsuppressiven Behandlung nach der Impfung.
‐ Im Allgemeinen können Totimpfstoffe bei Patienten, die eine systemische immunsuppressive Therapie erhalten, sicher verwendet werden. Jedoch kann die Immunogenität des Impfstoffs verringert sein. Lebendimpfstoffe sollten gemäß den jeweiligen Fachinformationen vermieden werden. Lebendimpfstoffe sollten auch bei Säuglingen (bis zu sechs Monate alt), deren Mütter nach der 16. Schwangerschaftswoche eine biologische Therapie erhalten haben, vermieden werden (siehe produktspezifische Fachinformationen und Kapitel zur Schwangerschaft).

‐ Es werden eine vollständige COVID‐19‐Impfung einschließlich einer zusätzlichen (dritten) Grunddosis und Auffrischungsimpfungen gemäß den nationalen Impfrichtlinien empfohlen, da Personen, die immunsuppressive Therapien erhalten, möglicherweise eine abgeschwächte humorale und zelluläre Reaktion auf den COVID‐19‐Impfstoff im Vergleich zu gesunden Personen haben.^27^, ^28^, ^29^ Die Unterbrechung einer Methotrexat‐Therapie von 2 Wochen nach einer Impfung sollte, sofern möglich, in Betracht gezogen werden, da dies die Immunogenität des Impfstoffs verbessern kann. In Studien ist der positive Einfluss dieser Maßnahme auf die klinische Wirksamkeit von Impfstoffen allerdings nicht erwiesen.^30^, ^31^ Es gibt keinen Konsens darüber, ob ein Verzicht auf Methotrexat den Schutz vor einer Infektion erhöht oder die Wahrscheinlichkeit für eine symptomatische Krankheit oder einen schweren COVID‐19‐Verlauf reduziert.

## HINWEIS ZUR LEITLINIENADAPTATION

Beim Verfassen dieser Leitlinie haben die Autoren*innen auf einer Vorversion der folgenden Publikation aufgebaut und diese außerdem adaptiert, neu zusammengesetzt und übersetzt: EUROGUIDERM GUIDELINE ON THE SYSTEMIC TREATMENT OF PSORIASIS von Nast A et al., deren finale Fassung auf der Webseite des European Dermatology Forum (https://www.guidelines.edf.one/guidelines/psoriasis-guideline) zur Verfügung steht (lizenziert unter CC BY NC 4.0, https://creativecommons.org/licenses/by-nc/4.0/):

- A Nast, PI Spuls, C Dressler, Z Bata‐Csörgö, I Bogdanov, H Boonen, EMGJ De Jong, I Garcia‐Doval, P Gisondi, D Kaur‐Knudsen, S Mahil, T Mälkönen, JT Maul, S Mburu, L Mercieca, U Mrowietz, A Pennitz, E Remenyik, D Rigopoulos, PG Sator, M Schmitt‐Egenolf, M Sikora, K Strömer, O Sundnes, G Van Der Kraaij, N Yawalkar, C Zeyen, C Smith. EUROGUIDERM GUIDELINE FOR THE SYSTEMIC TREATMENT OF PSORIASIS VULGARIS September 2023, partial update February 2025.

Darüber hinaus basieren die Inhalte des hier vorliegenden Artikels auf einer Adaptation der Vorversionen der Leitlinie, die in ihrer finalen Form wie folgt publiziert wurde:

- Nast, A., Altenburg, A., Augustin, M., Boehncke, W.‐H., Härle, P., Klaus, J., Koza, J., Mrüowietz, U., Ockenfels, H.‐M., Philipp, S., Reich, K., Rosenbach, T., Schlaeger, M., Schmid‐Ott, G., Sebastian, M., von Kiedrowski, R., Weberschock, T. and Dressler, C. (2021), Deutsche S3‐Leitlinie zur Therapie der Psoriasis vulgaris, adaptiert von EuroGuiDerm Teil 1: Therapieziele und Therapieempfehlungen. JDDG: Journal der Deutschen Dermatologischen Gesellschaft, 19: 934‐951. https://doi.org/10.1111/ddg.14508_g
- Nast, A., Altenburg, A., Augustin, M., Boehncke, W.‐H., Härle, P., Klaus, J., Koza, J., Mrowietz, U., Ockenfels, H.‐M., Philipp, S., Reich, K., Rosenbach, T., Schlaeger, M., Schmid‐Ott, G., Sebastian, M., von Kiedrowski, R., Weberschock, T. and Dressler, C. (2021), Deutsche S3‐Leitlinie zur Therapie der Psoriasis vulgaris, adaptiert von EuroGuiDerm Teil 2: Therapiemonitoring, besondere klinische Situationen und Komorbidität. JDDG: Journal der Deutschen Dermatologischen Gesellschaft, 19: 1092‐1117. https://doi.org/10.1111/ddg.14507_g

Die vorliegende adaptierte Leitlinie hat das Freigabeverfahren des European Dermatology Forum nicht durchlaufen, sondern wurde von den herausgebenden deutschen Fachgesellschaften freigeben. Diese Leitlinie unterliegt den Bestimmungen der Creative Commons Attribution‐NonCommercial.

## DANKSAGUNG

Open access Veröffentlichung ermöglicht und organisiert durch Projekt DEAL.

## INTERESSENKONFLIKT

Für die Autoren der deutschen Version: Siehe Leitlinienreport der deutschen Adaption auf https://register.awmf.org/de/leitlinien/detail/013-001. Für die Autoren der EuroGuiDerm Version: Siehe Methods Report: https://www.guidelines.edf.one/guidelines/psoriasis‐guideline [accessed: 10.04.2025])
